# Factors Influencing the Control of Hypertension According to the Gender of Older Adults

**DOI:** 10.3390/healthcare11111595

**Published:** 2023-05-30

**Authors:** Hye Young Choi, Eunha Kim

**Affiliations:** 1 Department of Nursing, Kangwon National University, Samcheok 25949, Republic of Korea; choihy3@kangwon.ac.kr; 2 Department of Nursing, Catholic University of Pusan, Busan 46252, Republic of Korea

**Keywords:** aged, hypertension, obesity, education

## Abstract

(1) Background: This study aimed to identify factors associated with hypertension control among older adults with hypertension based on their socio-demographic and health characteristics. (2) Methods: The sample consisted of a total of 1824 with hypertension and was obtained from the Eighth Korean National Health and Nutrition Examination Survey (VIII-1, VIII-2). (3) Results: As the factors associated with hypertension control among older men, 65–74 years old (OR = 1.76, CI = 1.04–2.96), a lower education level (OR = 2.23, CI = 1.17–4.28), with obesity (OR = 2.05, CI = 1.13–2.05), and under-treatment of hypertension (OR = 22.07, CI = 6.54–7.45) increased the likelihood of rating hypertension control. As the factors associated with hypertension control among older women, trying to weight maintain (OR = 1.70, CI = 1.01–2.85) and under-treatment of hypertension (OR = 12.16, CI = 3.65–40.46) increased the likelihood of rating hypertension control. (4) Conclusion: The factor affecting the control of hypertension differed between the two genders. To improve the control of hypertension, the guidelines for treatment interventions should be gender-specific for the early elderly. There is a need to increase control of hypertension by having health-related behavioral modifications such as reducing obesity for older men and trying weight maintenance for older women.

## 1. Introduction

### 1.1. Backgrounds

Korea’s older adult population is currently 16.5% of the total population and is expected to surpass 20% within 10 years [1]. The need for social support for older adults is imperative to prevent social isolation and deaths from chronic disease [2]. For instance, hypertension is a significant public health issue, and its incidence increases significantly with age. Among circulatory diseases worldwide, hypertension was the highest, ranked second for men and first for women in 2017 [3]. As the population of older adults increases, the prevalence of hypertension follows. In 2021, the prevalence rate of hypertension among adults over the age of 30 in Korea reached 30%. For people over the age of 65, it rose to 66% for men and 51% for women [4]. Since hypertension gradually causes arteriosclerosis and damages blood vessels, it can lead to cerebrovascular disease and ischemic heart disease. Therefore, controlling blood pressure is most important to reduce the mortality rate from the disease [5]. In 2019, the National Institute for Health and Care Excellence (NICE) published Hypertension Guidelines which stipulated lifestyle adjustments and antihypertensive drug therapy for older adults to control their blood pressure [6].

Previous studies in older adults with hypertension have reported associations with sociodemographic factors [7,8,9], health-related behavior [10], and obesity [11]. In the United States, where hypertension awareness, treatment, and control are the most prominent, only 50% of older adults aware of hypertension were receiving some kind of treatment. In addition, the control rate of older adults in their 80s decreased or stagnated by 30% [5]. Antihypertensive drug therapy for older adults over 80 years of age must account for frailty and comorbidities such as diabetes, dementia, heart failure, cancer, postural hypotension, and senile syndrome [6,12]. Other studies [8,11,13] have suggested that controlling hypertension differs by gender. Gender differences should be considered when planning for the future [14]. However, few studies have examined factors aside from age and sex that affect hypertension among older Koreans [8,13]. Therefore, more research is needed in this area. 

When analyzing the differences between gender and hypertension control among older adults in Korea, the previous studies [7,13] using a large sample suggested that a gender-sensitive approach can be effective in controlling high blood pressure among older adults. Additionally, in the study [7] using data from the Korean National Health and Nutrition Examination Survey (KNHANES), the subjects of the study were adults aged 30 or older, and the hypertension treatment and control were analyzed. These studies presented some limitations, such that they did not cover diverse variables for controlling hypertension among older adults. In earlier studies [7,13], selected variables reflect the treatment status according to a hypertension diagnosis in older adults. The present study, in turn, was conducted on older adults who are currently receiving medication or non-drug treatment. 

Factors associated with controlling hypertension, including demographic factors, socio-economic factors, health-related factors, health behavior, obesity, nutritional habits, and the recognition of hypertension are considered in this study using a sample of older participants. Thus, this study aimed to analyze the factors related to controlling hypertension among older adults, according to sex, with a focus on socio-demographic characteristics and health-related variables, ensuring reliability and representativeness by using data from the 8th KNHANES. To achieve adequate blood pressure of older adults, manage health care expenses efficiently, and ultimately provide basic data for preparing policies to improve the health of older adults with hypertension, we sought effective and economic measures.

### 1.2. Study Objectives

The aim of this study was twofold: (1) to identify the differences in controlling hypertension in older men and in older women based on their socio-demographic and health-related characteristics, and (2) to identify the factors associated with controlling hypertension based on these characteristics.

## 2. Materials and Methods

### 2.1. Study Design

This study employed a descriptive survey to examine sociodemographic and health-related characteristics associated with hypertension control among older adults aged 65 and over. Additionally, a secondary data analysis study was conducted using data from the Korea National Health and Nutrition Survey (KNHANES).

### 2.2. Study Participants

This study used raw data from the 8th 1st and 2nd year (2019–2020) KNHANES conducted by the KCDCP. The total number of respondents was 15,469 [15]. Of these, 3447 older adults were classified as participants in the first stage. In the second stage, 2103 people who attended the health, nutrition, and examination surveys were selected from 2121 older adults who had hypertension (systolic pressure ≥ 140 mmHg or diastolic pressure ≥ 90 mmHg or taking antihypertensive medication). In total, after those with missing values were excluded, 1824 people were classified as the final sample. Data on demographic and sociological characteristics, health status, and high blood pressure control were analyzed for 717 men and 1107 women. An adequate sample was considered to be at least 393 in each group using the G*Power 3.1 with an odds ratio of 1.5, a significance level of 0.05, and a power of 0.95.

### 2.3. Variables

The independent variables consisted of sociodemographic and health-related characteristics, and the dependent variable was whether their hypertension was controlled. 

#### 2.3.1. Hypertension Control Status

According to the 8th KNHANES raw data utilization guidelines [15], those with systolic blood pressure less than 140 mmHg and diastolic blood pressure less than 90 mmHg were defined as hypertension regulators and others as hypertension non-regulators based on the final value of blood pressure measured from the KNHANES data collection process.

#### 2.3.2. Socio-Demographic Characteristics

Age, education level, income level, and job status were identified as demographic and sociological characteristics. Participants’ age was classified into “65 to 74 years” and “75 years or older.” The level of education was divided into “elementary school graduate and below” and “middle school graduate and above.” Income level was divided into “lower” and “lower-middle or higher” based on the quartile of household income, and job status was divided into “employed” and “no or unemployed”.

#### 2.3.3. Health Related Characteristics

Alcohol consumption, current smoking status, aerobic physical activity practice, sedentary lifestyle, dietary control, weight control type, physical examination, perceived stress, obesity, restriction in daily function, hypertension recognition, and hypertension treatment were identified as health-related characteristics.

The amount of alcohol consumed at a time was divided into an average of three or more glasses and two or fewer glasses according to the classification of high-blood-pressure-dangerous alcohol [15]. Smoking status was divided into “smoker” and “non-smoker” at the time of the survey. Following the 8th KNHANES raw data usage guidelines for aerobic physical activities, participants were classified as practitioners if they practiced medium-intensity physical activities for more than 150 min a week, high-intensity physical activities for more than 75 min, or mid-mixed-high-intensity physical activity (1 min of high-intensity physical activity equals 2 min of mind-intensity physical activity). Sitting hours were divided into less than eight hours and more than eight hours based on the average daily sitting hours of older adults in Korea [16]. Dietary control was divided into “yes” and “no” for special reasons. Types of controlling weight were divided into “weight loss control,” “weight maintenance control,” “weight gain control,” and “no weight control” based on responses to a question on whether they had tried to control their weight on their own over the last year. Whether or not a physical examination had been conducted in the last two years was classified into “Yes” or “No.” Perceived stress was measured with four responses to one question, “How much stress do you feel in your daily lives?” and responses were classified as “a lot” or “a little.” Obesity status was classified as “obese” for a BMI of 25 kg/m^2^ or higher and others were classified as “not obese” based on the World Health Organization’s (WHO) Asian obesity standard [17]. Restrictions in daily functions were also obtained and the participants’ responses were divided into two categories, “yes” and “no,” for the question “Are your activities currently restricted in daily life and social activities due to health problems or physical or mental disorders?”. According to the 8th KNHANES raw data utilization guidelines [15], three representative indicators were defined for hypertension management. First, hypertension recognition was classified as “hypertension recognized” for those who responded to be diagnosed with hypertension by doctors, and as “not hypertension recognized” for the rest. Second, hypertension treatment was classified as “under hypertension treatment” for those who responded as currently taking hypertension drugs for 20 days or more per month and as “non-under hypertension treatment” for the rest. Third, hypertension control was explained above as an independent variable. 

### 2.4. Data Collection

During the KNHANES, two-stage stratified cluster sampling methods with enumeration districts and households as primary and secondary sampling were applied to ensure representativeness. The health and examination surveys were conducted in a mobile examination vehicle, and the nutrition survey including dietary control was conducted by visiting the target household in person. Education and economic activities, disease morbidity, and medical-use items in the health and nutrition survey were investigated via an interview, in addition, health behavior such as smoking and drinking in the health survey were investigated by self-report. In the case of cognitive impairment, the proxy persons participated in the interview together and answered the questionnaires. The vulnerable individuals without proxy persons were inevitably excluded from the study sample. The physical examination survey was measured directly, and blood pressure measurements were performed by four nurses at the Korean Centers for Disease Control and Prevention by applying blood pressure measurement standards and an investigator certification system. Blood pressure was measured three times at 30-s intervals after the participant had been seated for at least 5 min and the average value of the second and third rounds was used as the final blood pressure.

### 2.5. Ethical Considerations

Anonymized raw data were collected to ensure the confidentiality of the households and individuals that were surveyed following the predetermined procedures such as consenting to the use of personal information through the KNHANES website “https://knhanes.kdca.go.kr (accessed on 14 July 2022)”. The study was conducted according to the guidelines of the Declaration of Helsinki and its future amendments. Since this was secondary data analysis study, it was exempt from approval from the institutional review board of the Kangwon National University was obtained (approval number KWNUIRB-2023-01-008).

### 2.6. Data Analysis

Composite sample analysis was applied according to the ‘KNHANES Raw Data Analysis Guide’ [15] using SPSS/Win 24.0. Participant’s socio-demographic characteristics and health-related characteristics were calculated as weighted percentages, means, and standard errors. Difference in whether hypertension was controlled according to the population and participant’s sociological characteristics and health-related characteristics were examined via a complex sample *t*-test and χ^2^-test. Multiple logistic regression based on complex sample analysis was applied to identify factors related to the control of hypertension according to socio-demographic and health-related characteristics; statistical significance levels were *p* < 0.05.

## 3. Results

### 3.1. Difference in Control of Hypertension According to Socio-Demographic and Health-Related Characteristics in Older Adults with Hypertension 

#### 3.1.1. Difference in Control of Hypertension According to Socio-Demographic and Health-Related Characteristics in Older Men with Hypertension

The hypertension control rate among participants was 57.6%, of which, 63.6% was the rate for men. There was a statistically significant difference among participants according to age (χ^2^ = 11.89, *p* = 0.002), dietary control status (χ^2^ = 10.78, *p* = 0.003), weight control type (χ^2^ = 13.45, *p* = 0.026), obesity status (χ^2^ = 13.82, *p* = 0.001), recognition of hypertension (χ^2^ = 218.22, *p* < 0.001), hypertension treatment status (χ^2^ = 242.62, *p* < 0.001), and duration of hypertension (t = −2.15, *p* = 0.032) (Table 1). Specifically, the proportion of hypertension regulators was higher than that of non-controlled people among those aged 65–74, the diet control group, the weight loss and obese groups, the hypertension aware group, and the hypertension treatment group, and with a shorter prevalence period.

#### 3.1.2. Difference in Control of Hypertension According to Socio-Demographic and Health-Related Characteristics in Older Women with Hypertension

The hypertension control rate among older women was 53.0%. There were statistically significant differences among participants according to sitting hours (χ^2^ = 7.54, *p* = 0.020), obesity status (χ^2^ = 107.29, *p* < 0.001), recognition of hypertension (χ^2^ = 218.22, *p* < 0.001), and hypertension treatment status (χ^2^ = 242.62, *p* < 0.001) (Table 2). Specifically, the proportion of hypertension regulators was higher than that of non-controlled people in the group with more than eight hours of sitting per day, the obese group, those aware of hypertension, and the group undergoing hypertension treatment.

### 3.2. Factor That Influenced the Control of Hypertension According to Socio-Demographic and Health-Related Characteristics in Older Adults with Hypertension

Multiple logistic regression analysis was performed by introducing all independent variables to analyze the factors related to the control of hypertension in older adults with high blood pressure. Here, 16 variables, including age, education level, income level, job status, alcohol consumption, current smoking status, aerobic physical activity practice, sedentary lifestyle, diet control, weight control type, physical examination, perceived stress, obesity, activity restriction, and hypertension treatment were entered as dummy variables and one variable of hypertension prevalence duration was entered as a continuous variable.

#### 3.2.1. Factors That Influence the Control of Hypertension According to Socio-Demographic and Health-Related Characteristics in Older Men with Hypertension

The regression model in which socio-demographic and health status variables of older men were introduced was statistically significant (*p* < 0.001). It showed a 19% explanatory power. Age, education level, job status, alcohol consumption, obesity status, activity restriction, and hypertension treatment were significantly related factors for recognition of hypertension. Those with zero non-hypertension recognition among hypertension regulators were excluded from the analysis. Hence, the statistical values could not be presented (Table 3). Specifically, the group aged 65–74 years had 1.76 times more hypertension regulators than the group aged 75 or older (OR = 1.76, CI = 1.04–2.96). There were 2.23 times more hypertension regulators in the group with elementary school graduation or below than in the group with middle school graduation or above (OR = 2.23, CI = 1.17–4.28). The group with a job had 41% less hypertension control than the group without a job (OR = 0.59, CI = 0.34–0.96).

There were 43% fewer hypertension regulators in the group where participants consumed three or more glasses of alcohol compared to the group with two or fewer drinks (OR = 0.57, CI = 0.34–0.95), and 2.05 times more hypertension regulators in the group with obesity compared to the group without obesity (OR = 2.05, CI = 1.13–3.71). There were 55% fewer hypertension regulators in the restricted group compared to the group with activity restriction (OR = 0.45, CI = 0.22–0.91), and 22.07 times more hypertension regulators in the treated group compared to the non-hypertension treatment group (OR = 22.07, CI = 6.54–74.50).

#### 3.2.2. Factors That Influence the Control of Hypertension According to Socio-Demographic and Health-Related Characteristics in Older Women with Hypertension

The regression model in which socio-demographic characteristics and health-related variables of older women with hypertension were introduced was statistically significant (*p* ≤ 0.001). It showed a 10% explanatory power. Sedentary living time, weight control type, and hypertension treatment were identified as significant factors. For recognition of hypertension, those with zero non-hypertension recognition among hypertension regulators were excluded from the analysis and not presented (Table 3). Specifically, there were 37% fewer hypertension regulators in the group with less than 8 h compared to the group with more than 8 h of sedentary time per day (OR = 0.63, CI = 0.43–0.93), and 1.70 times more hypertension regulators in the group with weight maintenance efforts compared to the group without control attempt (OR = 1.70, CI = 1.01–2.85). In addition, there were 12.16 times more hypertension regulators in the group that underwent treatment than in the group that did not receive hypertension treatment (OR = 12.16, CI = 3.65–40.46).

## 4. Discussion

This study utilized data from the KNHANES (2019–2020) and identified the differences in hypertension control according to socio-demographic characteristics and health-related variables in older adults in Korea and analyzed factors related to hypertension control of older adults. Among the participants, 63.6% of men and 53.0% of women successfully controlled hypertension. This was consistent with previous results [11]. In other studies [18,19], older adults showed a lower control rate of hypertension in women than in men, which was similar to the results of this study. In contrast, one study [7] that analyzed the occurrence and control of high blood pressure using the KNHANES (2010–2014) showed that high blood pressure control in women was higher than in men. These results suggest that significant differences in hypertension control depend on gender. Furthermore, while the prevalence of hypertension gradually increased and cardiovascular disease was likely to occur due to physical changes such as blood vessel stiffness after menopause, the achievement of targeted blood pressure control through medication was low [20]. In addition, because the control of hypertension in older adults reflects physical changes according to aging, further study is required to identify the social and health-related characteristics that affect the control of hypertension according to the gender and age of older adults.

Hypertension control was 1.76 times higher in older men aged 65 to 74 years than in those aged 75 years and older. After the control of hypertension was examined based on age in 2111 older men and women in Canada [18], the control of hypertension for people in their 60 s was higher than that of those in their 70 s, which was partially consistent with the results of this study. Other studies of older adults in Ireland [19] and Germany [9] also support the results of this study. These studies were a cross-sectional analysis of the control rate of hypertension in older adults that decreased with age. Therefore, it was thought that the control of hypertension in older adults was related to a decline in physical function. Although this study did not select variables related to medication treatment in older patients with uncontrolled hypertension, the European Society for Hypertension [21] adjusted the treatment guidelines in 2018 to prescribe three or more antihypertensive drugs in combination for older adult patients whose target blood pressure was not achieved. Despite these efforts, resistance to enhanced drug therapy and side effects due to multiple drugs have hindered the implementation of drug use [18]. To control hypertension in older adults, it is necessary to develop and combine non-pharmaceutical therapy [20] that takes into account physical changes and declines in physical functions as we age.

Regarding education level, the control of hypertension in below elementary school graduates was 2.23 times higher than that of middle school graduates. Similar to this study, a study on hypertension control in those over 40 years in China [22] reported that elementary school graduates had 0.76 times lower than middle school graduates and 0.67 times than high school graduates. According to previous studies [9,23], the higher the level of education, the lower the risk of hypertension. A previous study [24] showed that the higher the level of education, the higher the failure to take hypertension drugs, as the criteria for participant selection included taking hypertension drugs, and 99.5% of male hypertension regulators received treatment. Cho et al. [24] used the KNHNAES (2008) to analyze the transition of drug use according to the educational level of older adults with high blood pressure, with elementary school graduates being the highest followed by middle school and high school graduates. These results suggest that it was necessary to differentiate strategies for controlling high blood pressure according to the educational level of older adults. Thus, the implementation of high blood pressure medication should be taught at the middle school or higher level. In addition, low-level seniors should be educated on screening activities, such as recognizing appropriate blood pressure, measuring blood pressure, and lowering blood pressure without the use of drugs.

Regarding job status, hypertension control was 41% lower in older men than in the absence of a job. Furthermore, according to an analysis of 653 people aged 60 years or older in Korea [11], the proportion of hypertension regulators in older men with jobs was higher than that in this study. Although the employment rate of older adults in Korea is high, the quality of jobs is poor. In addition, 44% of older adults employed had an average monthly income of less than 300,000 won, and 73% were engaged in agriculture, forestry, and fisheries. In addition, although expenses and cleaning workers were heavy part-time workers, many did not receive the minimum wage. Furthermore, the income level of the employed older adults was low owing to employment instability such as one to three months of ultra-short-term labor contracts [25]. Compared to older adults who worked long hours in poor working conditions, older adults without a job could participate in community-based hypertension management projects and gain the opportunity to improve their self-management in controlling their blood pressure [26]. However, it was believed that employed older adults had less time to access regular medical institutions and less motivation for exercise or dietary control, which leads to a lower level of hypertension control. Therefore, old-age occupations seem to hinder regular checkups and the practice of blood pressure control. However, in light of previous studies [11,18], it is necessary to confirm the difference in high blood pressure control according to the job.

The amount of drinking decreased blood pressure control 0.57 times in older men who consumed three or more glasses of alcohol, which supports the results of alcohol consumption in older men being harmful to controlling hypertension [7]. High-risk drinking with more than three drinks per week gradually decreased with age; however, the high-risk drinking rate of older men in Korea reached 45.4%, and drinking habits did not improve even after a hypertension diagnosis [27]. In Korean society, excessive drinking or one-time high-risk drinking for socializing is often tolerated, and drinking accompanied by meals is frequent, which can increase blood pressure and further the effectiveness of antihypertensive drugs [28]. Education should therefore emphasize the risks associated with drinking and drug interactions, encourage a reduction in alcohol consumption, and encourage the development of habits unassociated with eating and drinking.

Weight control status was an influential factor in the control of high blood pressure in older women, and the control of high blood pressure was 1.70 times higher in maintaining weight than not controlling it. This was consistent with the results of previous studies [7,9,18], which found that the control rate of hypertension among female seniors who maintained a normal weight was higher than that of overweight or obese older people. The WHO emphasized [29] that low-intensity exercise and physical activity were essential to reduce the incidence of chronic diseases which included high blood pressure in older adults. When a patient with hypertension lost 5 kg of weight, systolic blood pressure, and diastolic blood pressure were lowered by 4.44–3.57 mmHg on average [23]. In this study, hypertension control was higher in older women who remained sedentary for more than 8 h. It was estimated to be more dependent on medication than non-pharmaceutical approaches such as exercise and physical activity because it was not desirable for control of blood pressure through weight control in older adults. Due to aging, physical activity is limited due to reduced muscle strength, endurance, agility, and flexibility. In addition, the preference for a sedentary lifestyle over other leisure activities increased the risk of cardiovascular disease and type 2 diabetes [29]. Therefore, it is necessary to develop a community-based physical activity support program that reduces sedentary time and increases opportunities for low- or moderate-intensity activities in older people with high blood pressure.

Obesity status was an also influential factor in the control of hypertension in older men, and hypertension control was 2.05 times higher in older men than with obesity, which was consistent with previous studies [11,26] that analyzed the control rate of hypertension by gender in older men. In older adults in the UK, the control rate of hypertension decreased by 0.82 times in those who were overweight and 0.73 times in those who were obese compared to those which a normal weight [18]. In older adults in Canada [10], the control rate of hypertension decreased by 0.97 times in those with obesity compared to those with a normal weight, which was different from the results of this study. 

There was a significant difference in the control of high blood pressure in older men according to whether an activity was restricted. The control of high blood pressure was 55% lower when there was no activity restriction. Based on a Brazilian study [28], control of hypertension was 1.05 times higher in older adults without activity restrictions. Activity restrictions caused by poor physical and cognitive function in older adults resulted in poor mobility, decreased mobility, the poor activities of daily living (ADL), and decreased instrument activities of daily living (IADL), which resulted in lower blood pressure regulation practices [30]. According to these results, the decrease in physical and cognitive functions of older adults restricted their activity and hindered their practice of blood pressure control. It is, therefore, necessary to take into account whether physical and cognitive functions have deteriorated and whether aging has restricted activities to effectively control hypertension in older adults.

Depending on hypertension recognition status, hypertension control was 22.07 and 12.16 times higher for older men and women, respectively, and there were more hypertension regulators in older adults who were aware of hypertension. In this study, the awareness rates of hypertension were 83% for older men and 82.6% for older women. The control rate was 63.6% for older men and 53% for women. In a previous study [30], the awareness of hypertension and control rate in older adults was 94%, which was higher than in 2016. In a study of older adults in Germany [10], 90% of older adults with hypertension recognition reported that they controlled blood pressure [5], which was similar to this study. As a result, whether older adults were aware of high blood pressure was a prerequisite for blood pressure control. Furthermore, if the older adults recognize their high blood pressure status, they were motivated to change into a positive lifestyle and actively undertake treatment [5,9,10]. Therefore, to increase the control rate of hypertension, it is necessary to help older adults recognize the risks, recommend regular blood pressure measurements to help them understand and assess their blood pressure status, and encourage and educate them to continue receiving medication and non-drug therapy after being diagnosed. This will help them maintain their target blood pressure, prevent complications, and reduce the mortality rate from cardiovascular diseases.

As a result of the differences in hypertension control according to dietary control, there was a significant number of hypertension controllers in the diet control group in older men, which was consistent with the results of a previous study [13]. According to a study that analyzed the dietary control of 105 older people with high blood pressure registered at health clinics in rural areas, older adults in the first and second stages of hypertension had higher sodium intake than those with normal blood pressure or pre-hypertension. However, the hypertension control rate decreased by 71% compared to those whose salt intake was low. In addition, high-quality diets, which included breakfast availability, consumption of mixed grains, proteins, fresh fruits and vegetables, and intake of saturated fatty acids, salts, and sugars, had a positive effect on hypertension control in older men [31]. These results suggest that a low-salt diet is an important factor in controlling hypertension in older men. Furthermore, a high-quality diet that considers nutrient balance rather than practice helped control hypertension. Therefore, dietary control for older adults with hypertension should be developed and applied as an intervention program to control blood pressure. This can be implemented through a high-quality diet by identifying factors that affect eating habits, which include sodium intake.

This study was significant because it identified socio-demographic and health-related characteristic variables according to the control of hypertension in men and women using the 8th KNHANES data. This included older adults living in South Korea. These strengths might make our study more representative of community-dwelling older adults with hypertension in South Korea and contribute to our understanding of hypertension control, including behavioral and physical health factors. A preventive approach at the primary health care level has been attempted to manage hypertension in older Koreans. Through a public campaign, accurate information on high blood pressure as a major risk factor for cardiovascular disease is provided, and the importance of achieving target blood pressure through drug treatment and healthy lifestyle management is recognized. The Korean Society for Hypertension has newly published guidelines for treating hypertension that were released in 2022. They recommend that the definition of hypertension is 140/90 mmHg or more and that treatment goals be maintained if there is a high risk and cardiovascular disease lowered to less than 130/80 mmHg [32]. Active efforts are being made to improve the recognition rate and treatment rate of hypertension and systematic management measures for older adults aged 60 or older and those in their 20s and 30s.

Our study has several limitations. First, blood pressure was measured at a single time point among all participants. Ambulatory blood pressure monitoring and repeated measurements are recommended to assess diurnal and day-to-day blood pressure variations and to exclude the influence of an observer bias and white coat’s effect [13]. Thus, we cannot exclude the possibility of misclassification for the above reasons. However, we were not able to collect detailed information on antihypertensive medication use (the number of drugs, the status of drug changes if the target blood pressure was not achieved, and postural hypotension), comorbidities (diabetes, cardiovascular disease, dementia, etc.), age-related frailty, and other influences on blood pressure control. We selected the variables by referring to guidelines reported in other studies [7,13] as a study using secondary data. Future studies should collect detailed data reflecting the latest hypertension guidelines to comprehensively understand hypertension control among older adults.

## 5. Conclusions and Recommendation

This study used data from the KNHANES (2019–2020) and attempted to identify factors related to the control of hypertension in men and women aged 65 years or older. Furthermore, we prepared differentiated strategies for the control of hypertension, and provided basic data for efficient management. The significant differences in socio-demographic and health-related characteristics accord to sex. Furthermore, significant differences in hypertension control were found in older men according to age, diet control, weight control type, obesity, recognition of hypertension, and duration of hypertension. In older women, there was no significant difference according to socio-demographic characteristics. Among health-related characteristics, there was a significant difference in hypertension control according to sedentary lifestyle, obesity, recognition of the diagnosis and risks, and the treatment of hypertension. As a result of logistic regression analysis of the factors related to the control of hypertension in older men and women, age, education level, job presence, alcohol consumption, obesity, activity limitation, and treatment of hypertension were influential factors. Furthermore, the explanatory power of these variables was 19%. In older women, sedentary lifetime, weight control type, and treatment of hypertension were identified factors related to hypertension control, and the explanatory power of these variables was 10%.

These results led to the following suggestions. First, treatment guidelines should consider the sex of older adults when managing hypertension in older adults. Second, the development of services that reflect population, social, and health-related characteristics such as education level, job presence, and obesity for the control of hypertension in older men is necessary. Third, hypertension control for older women requires an educational program centered on health care services and counseling on weight control and hypertension treatment. Fourth, to effectively control hypertension in older adults, it is important to accurately recognize one’s blood pressure status and receive treatment. Therefore, a policy approach is required to improve high blood pressure recognition and treatment rate. Furthermore, to explore factors related to hypertension control in older adults, a longitudinal follow-up study is necessary.

## Figures and Tables

**Table 1:** Differences in Hypertension Control among Older Men with Hypertension by Socio-demographic and Health Status Characteristics (*n* = 717).

Variables			Total	Hypertension Control among Older Men with Hypertension		*χ*^2^ or t	*p*
				Yes (*n* = 462)	No (*n* = 255)		
			*n* ^†^ (% ^‡^) or M ± SE ^§^	*n* ^†^ (% ^‡^) or M ± SE ^§^	*n* ^†^ (% ^‡^) or M ± SE ^§^		
Socio- demographics	Age	65–74	417 (58.0)	287 (62.8)	130 (49.5)	11.89	0.002
		≥75	300 (42.0)	175 (37.2)	125 (50.5)		
	Education level *	≤Elementary	243 (37.4)	170 (39.3)	73 (33.9)	1.81	0.256
		≥Middle school	400 (62.6)	249 (60.7)	151 (66.1)		
	Income level *	Low	297 (38.5)	197 (37.7)	100 (40.1)	0.40	0.614
		≥Mid-lower	420 (61.5)	265 (62.3)	155 (59.9)		
	Occupation	Yes	270 (41.8)	177 (41.2)	93 (42.8)	0.15	0.734
		No	372 (58.2)	240 (58.8)	132 (57.2)		
Health status	Drinking amount (per time)	≥3	337 (48.6)	214 (47.3)	123 (50.8)	0.81	0.466
		≤2	374 (51.4)	245 (52.7)	129 (49.2)		
	Current smoking *	Yes	124 (18.0)	77 (17.1)	47 (19.4)	0.57	0.499
		No	586 (82.0)	382 (82.9)	204 (80.6)		
	Aerobic physical activity * (for last week)	Yes	233 (36.9)	153 (37.7)	80 (35.5)	0.30	0.648
		No	409 (63.1)	266 (62.3)	143 (64.5)		
	Sedentary time	<8 h	268 (41.2)	174 (40.9)	94 (41.7)	0.05	0.846
		≥8 h	369 (58.8)	239 (59.1)	130 (58.3)		
	Dietary Control *	Yes	196 (28.1)	136 (32.3)	60 (20,8)	10.78	0.003
		No	517 (71.9)	322 (67.7)	195 (79.2)		
	Body Weight Control	Weight loss	195 (27.7)	143 (32.2)	52 (19.7)	13.45	0.026
		Weight stay	170 (24.8)	108 (24.0)	62 (26.1)		
		Weight gain	33 (4.4)	20 (4.2)	13 (4.8)		
		No	312 (43.1)	188 (39.6)	124 (49.5)		
	Physical exam	Yes	481 (73.8)	319 (75.1)	162 (71.4)	1.07	0.342
		No	165 (26.2)	102 (24.9)	63 (28.6)		
	Perceived stress	Much	77 (10.2)	56 (11.8)	21 (7.5)	3.33	0.090
		A little	632 (89.8)	403 (88.2)	229 (92.5)		
	Obesity	Yes	286 (40.5)	206 (45.7)	80 (31.4)	13.82	0.001
		No	418 (59.5)	249 (54.3)	169 (68.6)		
	Restriction in daily functions	Yes	85 (13.1)	56 (12.7)	29 (13.8)	0.17	0.710
		No	561 (86.9)	365 (87.3)	196 (86.2)		
	Hypertension recognition	Yes	616 (85.2)	462 (100.0)	154 (59.3)	218.22	<0.001
		No	101 (14.8)	0 (0.0)	101 (40.7)		
	Hypertension treatment	Yes	596 (83.0)	459 (99.5)	137 (54.2)	242.62	<0.001
		No	121 (17.0)	3 (0.5)	118 (45.8)		
	Duration of hypertension *	Mean ± SD	14.03 ± 0.52	12.87 ± 0.49	15.18 ± 0.93	−2.15	0.032

* Missing values were excluded; ^†^ Non-weighted sample size; ^‡^ Weighted %; ^§^ Weighted mean ± standard error.

**Table 2:** Differences in Hypertension Control among Older Women with Hypertension by Socio-demographic and Health Status Characteristics (*n* = 1107).

Variables			Total	Hypertension Control among Older Women with Hypertension		*χ*^2^ or t	*p*
				Yes (*n* = 595)	No (*n* = 512)		
			*n* ^†^ (% ^‡^) or M ± SE ^§^	*n* ^†^ (% ^‡^) or M ± SE ^§^	*n* ^†^ (% ^‡^) or M ± SE ^§^		
Socio- demographics	Age	65–74	586 (54.5)	315 (55.2)	271 (53.8)	0.23	0.678
		≥75	521 (45.5)	280 (44.8)	241 (46.2)		
	Education level *	≤Elementary	667 (68.7)	372 (71.0)	295 (66.2)	2.51	0.193
		≥Middle school	277 (31.3)	134 (29.0)	143 (33.8)		
	Income level *	Low	578 (49.1)	311 (48.6)	267 (49.6)	0.12	0.755
		≥Mid-lower	522 (50.9)	280 (51.4)	242 (50.4)		
	Occupation	Yes	285 (31.6)	154 (31.3)	131 (32.1)	0.07	0.822
		No	659 (68.4)	353 (68.7)	306 (67.9)		
Health status	Drinking amount (per a time)	≥3	69 (6.6)	39 (7.2)	30 (6.0)	0.59	0.499
		≤2	1009 (93.4)	542 (92.8)	467 (94.0)		
	Current smoking *	Yes	25 (2.4)	18 (3.1)	7 (1.7)	2.29	0.255
		No	1051 (97.6)	562 (96.9)	489 (98.3)		
	Aerobic physical activity * (for last week)	Yes	260 (28.1)	132 (25.8)	128 (30.6)	2.72	0.144
		No	681 (71.9)	373 (74.2)	308 (69.4)		
	Sedentary time	<8 h	333 (37.2)	164 (33.0)	169 (41.8)	7.54	0.020
		≥8 h	577 (62.8)	327 (67.0)	250 (58.2)		
	Dietary Control *	Yes	275 (23.0)	150 (24.1)	125 (21.6)	0.95	0.381
		No	828 (77.0)	442 (75.9)	386 (78.4)		
	Body Weight Control	Weight loss	288 (26.4)	164 (27.7)	124 (25.0)	4.23	0.394
		Weight stay	160 (15.5)	89 (16.4)	71 (14.4)		
		Weight gain	69 (7.4)	35 (6.2)	34 (8.8)		
		No	562 (50.7)	294 (49.7)	268 (51.7)		
	Physical exam	Yes	669 (72.4)	361 (74.0)	308 (70.5)	1.420	0.243
		No	282 (27.6)	150 (26.0)	132 (29.5)		
	Perceived stress	Much	232 (22.5)	131 (23.5)	101 (21.3)	0.74	0.463
		A little	842 (77.5)	448 (76.50	394 (78.7)		
	Obesity	Yes	455 (42.9)	262 (47.3)	193 (38.0)	9.47	0.011
		No	600 (57.1)	303 (52.7)	297 (62.0)		
	Restriction in daily functions	Yes	188 (17.5)	102 (17.5)	86 (17.4)	0.00	0.956
		No	764 (82.5)	409 (82.5)	355 (82.6)		
	Hypertension recognition	Yes	948 (85.1)	595 (100.0)	353 (68.4)	217.78	<0.001
		No	159 (14.9)	0 (0.00)	159 (31.6)		
	Hypertension treatment	Yes	919 (82.6)	590 (99.4)	329 (63.7)	245.55	<0.001
		No	188 (17.4)	5 (0.6)	183 (36.3)		
	Duration of hypertension *	Mean ± SD	14.00 ± 0.35	13.36 ± 0.40	14.64 ± 055	−1.93	0.054

* Missing values were excluded; ^†^ Non-weighted sample size; ^‡^ Weighted %; ^§^ Weighted mean ± standard error.

**Table 3:** Associated Factors of Hypertension Control by Gender (*n* = 1824).

	Variables		Hypertension Control among Older Men with Hypertension (*n* = 717)			Hypertension Control among Older Women with Hypertension (*n* = 1107)		
			OR	95% CI	*p*	OR	95% CI	*p*
Socio- demographics	Age	65∼74	1.76	1.04–0.96	0.035	1.27	0.84–0.92	0.252
		≥75	1					
	Education level	≤Elementary	2.23	1.17–0.28	0.015	1.07	0.70–0.63	0.768
		≥Middle school	1					
	Income level	Low	0.79	0.48–1.33	0.379	1.01	0.71–1.44	0.951
		≥Mid-lower	1					
	Occupation	Yes	0.59	0.37–0.96	0.032	0.96	0.63–1.49	0.853
		No	1					
Health status	Drinking amount (per time)	≥3	0.57	0.34–0.95	0.030	0.85	0.46–1.57	0.602
		≤2	1					
	Current smoking	Yes	1.54	0.75–3.13	0.237	3.18	0.99–10.23	0.052
		No	1					
	Aerobic physical activity (for last week)	Yes	1.44	0.83–2.50	0.195	0.94	0.62–1.42	0.767
		No	1					
	Sedentary time	<8 h	0.92	0.57–1.48	0.720	0.63	0.43–0.93	0.021
		≥8 h	1					
	Dietary Control	Yes	1.21	0.73–2.02	0.464	1.07	0.73–1.59	0.721
		No	1					
	Body Weight Control	Weight loss	0.90	0.46–1.75	0.755	1.13	0.74–1.75	0.567
		Weight stay	0.87	0.50–1.50	0.606	1.70	1.01–2.85	0.047
		Weight gain	0.68	0.20–2.32	0.535	1.25	0.60–2.62	0.547
		No	1					
	Physical exam	Yes	1.31	0.75–2.28	0.337	0.95	0.64–1.41	0.796
		No	1					
	Perceived stress	Much	1.54	0.70–3.38	0.280	1.18	0.77–1.79	0.453
		A little	1					
	Obesity	Yes	2.05	1.13–3.71	0.018	1.39	0.92–2.11	0.118
		No	1					
	Restriction in daily functions	Yes	0.45	0.22–0.91	0.027	0.83	0.55–1.26	0.385
		No	1					
	Hypertension Recognition *	Yes						
		No	1					
	Hypertension treatment	Yes	22.07	6.54–74.50	<0.001	12.16	3.65–40.46	<0.001
		No	1					
	Duration of hypertension		0.97	0.95–1.00	0.021	0.97	0.95–0.99	<0.001
	F (*p*)		4.10 (<0.001)			2.45 (0.001)		
	R^2^		0.19			0.10		

* The statistic value for ‘Hypertension recognition’ variable was not shown because the number of subjects who control hypertension but was not recognized their own hypertension disease was ‘zero’.

## Data Availability

The datasets used and/or analyzed during the current study are available from the corresponding author on reasonable request.
